# Clinical Outcomes and Complications of Dental Implants Placed and Restored by AEGD Residents: Up to 10-Year Retrospective Study

**DOI:** 10.3390/dj14030185

**Published:** 2026-03-23

**Authors:** Nisreen Al Jallad, Eli Sun, Ethan Hang, Radhika Thakkar, Neha Naik, Shasha Cui, Amer Basmaji, Tongtong Wu, Alexis Ghanem, Mohammed Baig, Jin Xiao, Hans Malmstrom

**Affiliations:** 1 Eastman Institute for Oral Health, University of Rochester School of Medicine and Dentistry, Rochester, NY 14620, USA; nisreen_aljallad@urmc.rochester.edu (N.A.J.); radhika_thakkar@urmc.rochester.edu (R.T.); neha_naik@urmc.rochester.edu (N.N.); shasha_cui@urmc.rochester.edu (S.C.); amer_basmaji@urmc.rochester.edu (A.B.); alexis_ghanem@urmc.rochester.edu (A.G.); bobbybaig@gmail.com (M.B.); jin_xiao@urmc.rochester.edu (J.X.); 2 Department of Biostatistics and Computational Biology, University of Rochester Medical Center, Rochester, NY 14620, USA; eli_sun@urmc.rochester.edu (E.S.); ethanhangwork@gmail.com (E.H.); tongtong_wu@urmc.rochester.edu (T.W.)

**Keywords:** dental implants, AEGD, implant survival, implant complications, patient satisfaction

## Abstract

**Background:** Implant therapy is a standard of care for long-term tooth replacement. While high survival rates have been reported for implants placed by specialists, data on outcomes achieved by Advanced Education in General Dentistry (AEGD) residents remain limited. **Objectives:** To evaluate the clinical performance and complication rates of dental implants placed and restored by AEGD residents under faculty supervision, and to identify factors influencing outcomes after at least one year in function. **Methods:** A retrospective review was conducted for implants placed between April 2012 and December 2021 at the Eastman Institute for Oral Health. Only implants with ≥1-year follow-up were included. Data included demographics, medical history, smoking status, oral hygiene, peri-implant health, and prosthetic outcomes. Logistic regression was used to assess associations between risk factors and complications. **Results:** Among 262 implants that survived ≥1 year, the complication rate was low: screw loosening occurred in 8.4%, crown issues in 3.4%, abutment or screw fractures in 0.4%, and early peri-implantitis in 11.5%. Examiner satisfaction was high for esthetics (82.8%) and occlusion (85.9%), and over 80% of patients rated their outcomes between 8 and 10 on a 10-point scale. Diabetes and high plaque index were significant predictors of peri-implantitis. **Conclusions:** Implants placed and restored by AEGD residents under structured faculty supervision achieved low complication frequencies, and strong patient satisfaction comparable to specialist outcomes.

## 1. Introduction

Dental implant therapy has become a widely accepted and predictable standard of care for the replacement of missing teeth, with long-term survival rates exceeding 90% in most clinical reports [1]. Multiple systematic reviews have demonstrated that implant-supported restorations provide excellent functional, esthetic, and patient-reported outcomes [2,3,4]. Despite these advances, the long-term success of implant therapy depends on clinician skill, treatment planning, surgical technique, and ongoing maintenance [5,6].

While high survival rates have been documented in specialty and private practice settings, limited data exists regarding outcomes achieved by general dentistry residents. Advanced Education in General Dentistry (AEGD) programs provide residents with valuable exposure to both surgical and prosthetic phases of implant therapy under faculty supervision [7,8].

The most recognized standard for defining implant success remains Albrektsson’s criteria [9], which outline ideal biological and clinical parameters for implant health. However, these represent optimal conditions and may not account for implants that remain functional despite minor biological changes. The 2010 Academy of Osseointegration consensus report broadened the definition by including implants with minimal bone loss and no progressive pathology as clinically successful [5]. Ultimately, implant success extends beyond osseointegration to the long-term maintenance of a stable, functional, and esthetically acceptable restoration that fulfills patient expectations.

Implant education has become an essential component of modern dental curricula. Mattheos et al. [10] emphasized integrating implant training across educational levels as implant therapy grows globally. Similarly, Petropoulos et al. [11] surveyed U.S. and Canadian dental schools and found that although 97% of programs provided didactic implant instruction, only 86% offered clinical experience, and most lacked competency requirements, often due to limited trained faculty. These findings highlight the need for structured postdoctoral programs such as AEGD residencies to ensure comprehensive, hands-on implant training.

Cumulative survival rates near 96% have been reported for implants placed by specialty resident–faculty teams, yet few studies have evaluated outcomes within general dentistry programs [12]. Starr and Maksoud [13,14] reported survival rates of 98.2% (6 months–4 years) and 96.6% (7 years) for implants placed by AEGD residents, comparable to outcomes achieved by experienced clinicians.

Evaluating the performance of implants placed and restored by AEGD residents provides valuable insight into the effectiveness of such programs and their contribution to clinical quality assurance. This study was designed to address this knowledge gap.

## 2. Objective

To evaluate the clinical outcomes and complication rates of dental implants placed and restored by residents under faculty supervision in the Advanced Education in General Dentistry (AEGD) program at the Eastman Institute for Oral Health, University of Rochester, NY, and the primary aim is to identify factors influencing implant outcomes after at least one year in function.

We hypothesize that implants placed by AEGD residents would demonstrate clinical outcomes comparable to those reported for specialists.

Secondary aims: To assess patient satisfaction, identify risk factors for implant failure or complications, and examine outcomes relative to medical and demographic variables.

## 3. Materials and Methods

### 3.1. Study Design, Population, and Data Collection

This retrospective chart review was conducted at the Eastman Institute for Oral Health (EIOH), University of Rochester, and approved as an exempt protocol by the University’s Research Subjects Review Board (RSRB STUDY00007894). Electronic dental records were reviewed for patients who received dental implants and final restorations by AEGD residents between 1 April 2012, and 31 December 2021. Recall examinations and related documentation were completed by calibrated residents using a standardized electronic template (EXAM11). Calibration was achieved through comparison with a gold standard established by the principal investigator (HM), who validated inter-examiner reliability across three independent assessments. Patient identification was facilitated using ADA procedure codes for implant-related procedures. All follow-up data were collected as part of standard clinical documentation, ensuring consistency, accuracy, and quality control throughout the study period.

### 3.2. Variables and Data Collection

Data extracted from patients’ electronic records (axiUm) included demographic variables (age, sex, race/ethnicity) and medical status classified according to the American Society of Anesthesiologists (ASA) system [13]. Systemic risk factors, smoking habits, oral hygiene, and frequency of dental visits were also recorded. Implant-related data included year of placement, use of grafting or sinus augmentation, implant type, size, shape, and level, as well as abutment material and restoration type. Clinical parameters evaluated were gingival index (Löe & Silness) [14], bleeding on probing, gingival biotype, gingival recession, keratinized tissue width, probing depth, and papilla index (Jemt) [15]. Radiographic bone loss, peri-implantitis, and implant–abutment connection type were also assessed. Prosthetic outcomes were evaluated using modified USPHS criteria [16], and both patient and provider satisfaction were recorded on a 0–10 Likert scale for esthetics, function, occlusion, and overall experience. Data were obtained from standardized recall notes (EXAM11), which included comprehensive soft- and hard-tissue assessments.

### 3.3. Characteristics of the Study Population

As part of routine care, patients receiving dental implants in the AEGD program at the Eastman Institute for Oral Health (EIOH) underwent recall examinations every six months for two years following delivery of the final restoration and annually thereafter. The study population comprised patients whose implant and restoration procedures were completed by AEGD residents between 1 April 2012, and 31 December 2021. Only adults (≥18 years) were included. Because of the retrospective design, randomization and blinding were not applicable.

**Inclusion criteria:** Dental charts of patients aged 18 years or older with complete electronic documentation (axiUm), including periapical radiographs before surgery, at implant placement, at prosthesis delivery, and during at least one recall visit ≥1 year post-restoration.

**Exclusion criteria:** Patients younger than 18 years, those with <1-year follow-up, or with incomplete radiographic or clinical documentation.

### 3.4. Criteria for Implant-Related Outcomes

Definition of implant success, survival and failure: Three primary categories were established by the International Congress of Oral Implantologists (ICOI) Pisa Consensus Conference [17]: success, survival, and failure. The success category describes optimum conditions, the survival category describes implants still in function but not with ideal conditions, and the failure of an implant represents an implant that should be or already has been removed.

Survival conditions for implants may have two different categories: satisfactory survival describes an implant with less-than-ideal conditions yet does not require clinical management; and compromised survival includes implants with less-than-ideal conditions, which require clinical treatment to reduce the risk of implant failure. Implant failure is the term used for implants that require removal or have already been lost [6].

Definition of prosthesis success: Prosthesis success will be defined as prosthesis with absence of technical complications and with adequate function and esthetics [18].

Definition of prosthesis survival: Prosthesis survival will be defined as prosthesis remaining in situ with or without modifications during the entire observation time [18].

Definition of prosthesis failure: A prosthesis failure is defined as an event leading to:

- Loss of the prosthesis.
- Need to renew the entire implant-supported prosthesis.
- Explantation/loss of the implant(s) and subsequent loss of the prosthesis [18].

Definition of peri-implantitis: According to the consensus report of the 2017 World Workshop on the classification of periodontal and peri-implant diseases and conditions, healthy peri-implant tissues are characterized by the absence of erythema, bleeding on probing, swelling, and suppuration. Peri-implant mucositis is then considered present when peri-implant soft tissues bleed on gentle probing while erythema, swelling, and/or suppuration may also be present. Peri-implantitis is defined as the presence of bleeding and/or suppuration on gentle probing, probing depths of >6 mm, and bone levels >3 mm apical to the most coronal portion of the intraosseous part of the implant [19].

Definition of Complication: Any prosthesis/implant having any of the following characteristics: biologic complications including radiographic bone loss, probing depth, bleeding index, and plaque index, peri-implant mucositis, peri-implantitis, soft tissue recession and/or dehiscence, peri-implant bone loss exceeding 2 mm after 1 year of functional loading, hypertrophy/hyperplasia of soft tissue, and late implant failure.

Prosthetic complications including de-cementation of crown on abutment, fracture and/or chipping of restorative material, fracture/chipping of opposing dentition, wear of the opposing dentition, loosening of implant crown/abutment, fracture of the implant or abutment, and screw fracture.

### 3.5. Criteria for Bone Loss

Several studies report a yearly radiographic marginal bone loss after the first year of function in the range of 0 to 0.2 mm [17]. Pisa Consensus suggests that the clinical assessment for each implant monitors marginal bone loss in increments of 1.0 mm. The bone loss measurement should be related to the original marginal bone level at implant insertion, rather than to a previous measurement (e.g., 1 year prior) [17].

### 3.6. Data Reporting

In this study, we assessed a range of clinical, demographic, and procedural variables from the electronic dental records of patients whose implants remained in situ for at least one year. As such, this dataset did not include implants that failed within the first year or were removed prior to follow-up. Consequently, a true time-to-event analysis of implant survival could not be conducted, and the study does not provide survival or failure rates in the traditional sense. Instead, we got data from administrative billing records indicated by the codes D6010 (Surgical placement, endosteal implant) and code D6100 (implant removal) during the study period. Our analysis focused on evaluating biological and prosthetic complications among functioning implants, as well as patient and provider satisfaction outcomes.

Complication rates were reported as proportions based on clinical findings documented in implant recall visits (EXAM11). Biologic complications were assessed by examining radiographic bone loss (mm), which was represented as a mean. Probing depth (mm), bleeding index (%), and plaque index (%), all continuous variables, were also represented as means. Biologic complications, such as peri-implant mucositis, peri-implantitis, soft tissue recession and/or dehiscence, peri-implant bone loss exceeding 2 mm after one year of functional loading, hypertrophy/hyperplasia of soft tissue, and late implant failure, were documented as counts to calculate the proportion of complications.

**Figure 1:**
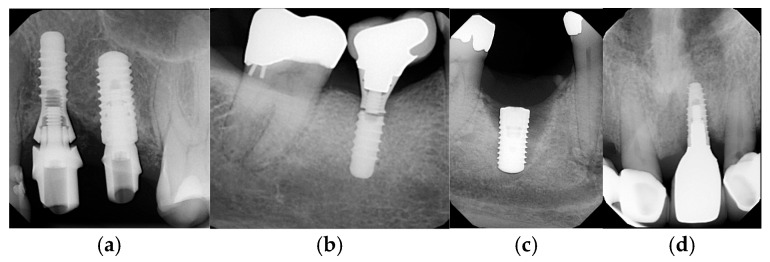
**Examples of implant-related complications:** Screw loosening (**a**), implant fracture (**b**), early implant failure 7 months postoperatively in a diabetic smoker patient (**c**), and late implant failure 4 years postoperatively (**d**).

Additionally, prosthetic complications, such as de-cementation of crowns on abutments, fracture and/or chipping of restorative material, loosening of implant crowns or abutments, fracture of the implant or abutment, and screw fractures, were recorded as counts to calculate their respective proportions Figure 1.

## 4. Results

### 4.1. Study Population and Demographics

A total of 262 implants placed in 131 unique patients met the inclusion criteria and were included in the evaluation. Most patients received one implant 62 (47.3%); 42 (32.1%) received two implants; and 27 (20.6%) received three or more implants. All implants were placed and restored by AEGD residents at the Eastman Institute for Oral Health (EIOH). These cases had at least one year of documented follow-up. The patients ranged in age from 20 to 89 years, with a mean age of 62.2 years (median: 62; SD: 12.9). The cohort was nearly balanced by gender (52.7% female). Most patients identified as White (58.4%), while 11.1% identified as Black, 2.7% as Asian, 2.3% as Native American; 14.5% declined to disclose their race and 11.1% were unspecified. Regarding ethnicity, 12.2% identified as Hispanic.

Commonly reported medical conditions included hypertension (28.6%), alcohol use (26.3%), and diabetes mellitus (22.9%). Arthritis was reported in 16.4% of patients, and 18.3% reported tobacco use. Notably, 18.3% of patients had no significant medical conditions. Penicillin allergy was the most frequently reported allergy (14.5%) Table 1.

### 4.2. Follow-Up Duration

The distribution of follow-up duration is presented in Table 2. The mean follow-up duration of the 262 implant was 3.75 years (SD, 1.92), with a median of 3.25 years (range, 0.67–9.25 years). Most implants (73.3%) were followed for less than 5 years, whereas only 9.2% had 7 or more years of observation.

### 4.3. Implant Retention Rates, Biological and Mechanical Complications

Administrative billing records indicated a 2.28% implant removal rate during the study period. Clinical complication rates were calculated separately among implants that remained in function at the time of recall examination.

Baseline periapical radiographs were obtained at the time of implant placement using the paralleling technique. Radiographic calibration was performed using the implant thread pitch to correct for distortion, with the implant platform serving as the fixed reference point. Marginal bone levels were measured at the mesial and distal surfaces from the implant platform to the first bone-to-implant contact using digital measurement tools. All radiographs were independently evaluated by calibrated examiners.

Among the 262 cases evaluated, 88.17% showed no signs of the condition. Early peri-implantitis (PD ≥ 4 mm, BOP, Bone loss < 25% of length) was observed in 11.45% of patients. Moderate peri-implantitis (PD ≥ 6 mm, BOP, Bone loss 25–50% of length) was rare, occurring in just 0.38% of cases and no case was recorded for severe peri-implantitis (PD ≥ 8 mm, BOP, Bone loss >50% of length). The “early,” “moderate,” and “severe” implantitis categories were defined based on the extent of crestal bone loss relative to the implant length. This grading system is widely used in clinical implant studies, though it has not been formally validated as a standardized scale [20].

Implant-related mechanical complications were infrequent: abutment and screw fractures were each reported in only 0.38% of cases, while screw loosening was the most common, affecting 8.39%. Regarding crown-related issues, loosening of the cement seal and chipping of the crown were each reported in 3.43% of cases, and crown fractures occurred in 0.76%. Qualitative assessments of the prosthesis showed high levels of success: marginal adaptation was rated as Alpha (ideal) in 93.89% of cases and as Bravo (minor deviation) in 6.11%. Anatomical form was ideal in 91.22% and slightly contoured in 8.78% of cases. Color match was ideal in 92.75%, slightly mismatched in 6.49%, and had a Charlie (major mismatch) in only 0.76%. Occlusal wear was rated as ideal in 99.24% of cases, with 0.38% showing minor or major wear, respectively. Overall, these outcomes indicate that the most frequent complication was early peri-implantitis (11.45%), followed by screw loosening (8.39%). Other complications occurred at rates below 4%. Table 3.

### 4.4. Patient-Reported Satisfaction

**Figure 2:**
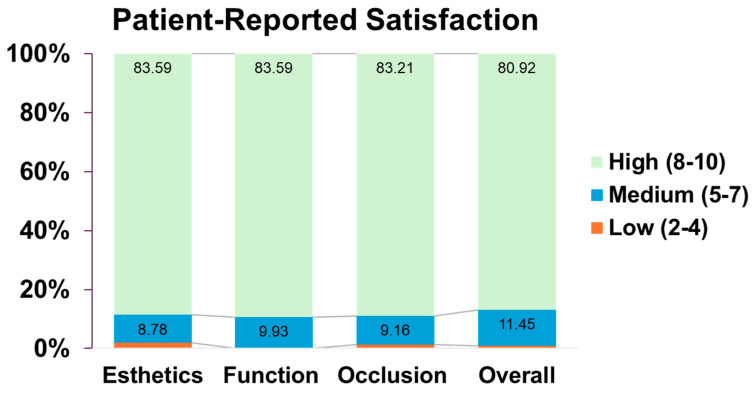
**Patient-reported satisfaction with implant outcomes across four domains (262 implants):** Distribution of patients’ satisfaction scores on 10-point Likert scale. Percentages based on available responses; 5.7–6.9% of data not available per domain.

Figure 2 illustrates that most patients rated their satisfaction in the high range (scores 8–10) in 83.59% of cases for esthetic and function, 83.21% for occlusion and 80.92% for overall experience. Medium satisfaction (scores 5–7) was less frequent but still notable, especially at score 7, which was selected by 7.25% for esthetics, 5.73% for function, 6.11% for occlusion, and 8.4% for overall satisfaction. Low satisfaction (scores 2–4) was rarely reported, with only 1.53% expressing low satisfaction with esthetics, and less than 1% in other categories. No patients reported scores of 0 or 1 in any domain. A small portion of data were not available (NA), ranging from 5.73% to 6.87% depending on the category.

### 4.5. Examiner-Reported Outcomes

**Figure 3:**
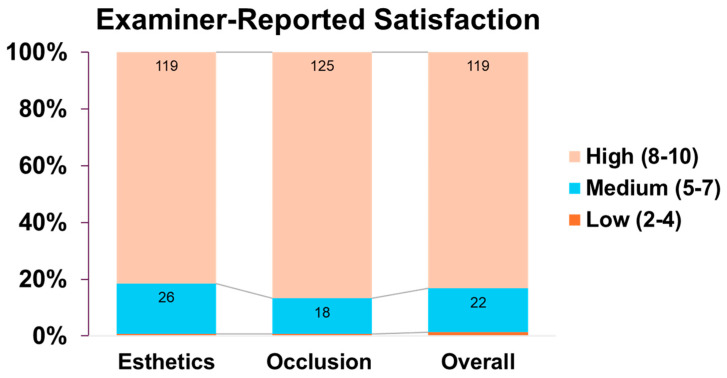
**Examiner-reported satisfaction with implant outcomes across three domains (262 implants)**: Distribution of examiner satisfaction scores on 10-point Likert scale. Percentages based on available responses; 6.87% of data not available per domain.

Provider evaluations using a 0–10 Likert scale demonstrated high satisfaction (scores 8 to 10) in 82.83% of cases for esthetics, 85.88% for occlusion, and 83.59% for overall performance. Moderate or neutral satisfaction (scores 5 to 7) was observed in 9.92% of cases for esthetics, 6.87% for occlusion, and 8.39% overall. Low satisfaction (scores 2 to 4) was rare, reported in only 0.38% to 0.76% of cases across all domains. Notably, only one case (0.38%) was classified as unsatisfied (score of 0 or 1), and 6.87% of responses were marked as not available (NA) across all categories. Figure 3.

### 4.6. Predictive Factors Related to Implant Outcome

Multivariable regression identified several key associations:

1. Predictive factors for outcomes when the implant has peri-implantitis: Holding all other variables constant, patients with diabetes had 3.8 times higher odds of peri-implantitis (OR 3.79, 95% CI) compared to those without diabetes. Additionally, each additional year of age was associated with a 4.7% reduction in the odds of peri-implantitis. In contrast, for each 1-unit increase in plaque index score, the odds of developing peri-implantitis increased by approximately 2.23 times, underscoring the strong association between plaque accumulation and peri-implant disease.
2. Predictive factors for implant-related complications (abutment, screw, crown fracture, screw loosening, loosening in cement): Holding everything else constant, odds of experiencing an implant-related outcome were 3.25 times higher for patients who had a bone grafted implant (OR 3.25, 95% CI) relative to those who did not.
3. Predictive factors for implant-crown-related complications (marginal adaptation, crown chipping, occlusal wear, color match and anatomical form): Holding everything else constant, each additional year of age is associated with an approximately 0.92 times change in the odds of experiencing an implant crown–related outcome, indicating that the odds decrease by about 8% for each one-year increase in age.

### 4.7. Predictive Factors Related to Patients’ Satisfaction Outcome

1. **Esthetic:** Patients receiving implants with irregular neck designs had markedly lower odds of reporting esthetic satisfaction, approximately 98% less likely than those with regular neck designs. In contrast, placement of a temporary crown significantly improved esthetic satisfaction, increasing the odds of total satisfaction by nearly eightfold. Age also showed a positive association, with each additional year corresponding to an approximate 9% increase in the likelihood of esthetic satisfaction.
2. **Function:** Adjusted analyses showed that White patients were over six times more likely to report satisfaction with implant function than other patients. Conversely, patients with hypertension had approximately 84% lower odds of total satisfaction. Each additional year of age increases the likelihood of satisfaction by about 6%. Patients who underwent sinus lift procedures were four times more likely to report functional satisfaction, whereas those receiving implants with irregular neck designs had about 95% lower odds of being fully satisfied with function.
3. **Occlusion:** After adjustment for covariates, patients who underwent sinus lift procedures had approximately 102-fold higher odds of reporting total satisfaction with occlusion, while those receiving temporary crowns had 46-fold higher odds. Conversely, irregular implant neck design, hypertension, and alcoholism were each associated with substantially lower satisfaction, reducing odds by about 97%, 85%, and 76%, respectively. White patients were nearly four-times more likely to report full satisfaction compared with other patients. Each one-unit increase in plaque index score corresponded to a 62% reduction in the odds of satisfaction with occlusion.
4. **Overall:** After adjusting for other variables, several factors were found to significantly influence overall patient satisfaction with implant treatment. Satisfaction was significantly higher among patients who underwent sinus lift procedures, received socket preservation, or had temporary crowns placed showing approximately 80-, 6-, and 15-fold greater odds of total satisfaction, respectively. Increasing age modestly improved satisfaction, with a 9% rise per additional year. In contrast, patients with hypertension, irregular implant neck designs, or higher plaque index scores reported markedly lower satisfaction, corresponding to 91%, 99%, and 76% reductions in the odds of full satisfaction, respectively.

## 5. Discussion

This retrospective study evaluated clinical performance, complication rates, and patient satisfaction associated with dental implants placed and restored by AEGD residents at the Eastman Institute for Oral Health (EIOH). Importantly, only implants that remained in situ for at least one year post-restoration were included in the analysis. Therefore, this study does not assess implant survival in the traditional sense but rather focuses on the long-term outcomes of functioning implants under real-world clinical conditions.

Implant success is commonly defined using the criteria originally proposed by Albrektsson et al., 1986 [9], which remain widely accepted in clinical research and practice. These include the absence of implant mobility, persistent pain, infection, neuropathies, or paresthesia; no evidence of peri-implant radiolucency on radiographs; marginal bone loss not exceeding 1.5 mm during the first year and no more than 0.2 mm annually thereafter; and the continued functional use of the implant in supporting a prosthesis [9].

Our results compare favorably with large-scale meta-analytic data. Jung et al. (2012) [21] systematically reviewed 46 studies comprising over one thousand implant-supported single crowns (SCs) and reported 5- and 10-year survival rates of 97.2% and 95.2%, respectively. The cumulative biological complication rates were 7.1% for soft tissue issues and 5.2% for bone loss > 2 mm, while technical complications reached 8.8% for screw loosening, 4.1% for loss of retention, and 3.5% for veneering fractures after 5 years. In comparison, implants placed by AEGD residents in our study demonstrated comparable and even lower complication frequencies: screw loosening occurred in 8.39% of cases, while abutment and screw fractures were observed in only 0.38% each, and crown-related issues such as chipping or cement loosening appeared in 3.43% of cases. No cases of severe peri-implantitis were recorded.

Our regression analysis identified diabetes as a significant predictor of peri-implantitis, with affected patients demonstrating approximately 3.8 times higher odds of developing peri-implant disease compared with non-diabetic individuals. This finding aligns with the systematic review and meta-analysis by Monje et al. (2017) [22], which concluded that diabetes mellitus or hyperglycemia is independently associated with an increased risk of peri-implantitis, regardless of smoking status, although not with peri-implant mucositis.

In the present study, smoking and poor oral hygiene were associated with higher odds of peri-implant complications, aligning with previous evidence demonstrating the detrimental effects of smoking on implant prognosis. Strietzel et al. (2007) [23] conducted a systematic review and meta-analysis including 29 studies and reported a significantly increased risk of implant failure among smokers, with implant-related odds ratios of 2.25 (95% CI: 1.96–2.59) and patient-related odds ratios of 2.64 (95% CI: 1.70–4.09) compared with non-smokers. The review also highlighted that smokers undergoing bone augmentation procedures exhibited an even greater risk of implant failure (OR 3.61; 95% CI: 2.26–5.77). These findings support our observations and emphasize the need for rigorous risk assessment and smoking cessation counseling before implant therapy.

These findings suggest that the outcomes achieved by residents under structured supervision closely mirror or even exceed those reported in long-term meta-analyses from specialist or mixed clinical populations. The low biological and technical complication rates observed further underscore the effectiveness of standardized protocols and faculty-guided training implemented in the AEGD program.

The findings demonstrate a low incidence of biological and mechanical complications and high levels of patient and provider satisfaction. These outcomes are encouraging and suggest that, when supported by structured training and close faculty supervision, general dentistry residents can successfully manage implant surgery to a standard comparable to that of specialized programs. Our findings align with earlier reports that dental residents can achieve excellent implant success rates. For instance, a University of Florida study of first-year general dentistry residents reported a 98% implant survival rate—surpassing the 90–95% success commonly seen with experienced clinicians—after 279 implants over a 4-year period [24]. However, because failed and removed implants were excluded, the survival rate itself cannot be inferred from our study.

Another factor to consider is that our analysis included only implants that survived and remained in situ at the time of recall. Severe peri-implantitis often leads to early implant removal, which would exclude such cases from our dataset. This may explain why no severe peri-implantitis was documented in our cohort, as implants with advanced disease could have been removed before the recall visit and therefore not captured in this review.

A particularly noteworthy aspect of this study is the observed influence of sociodemographic and procedural factors on satisfaction levels. White patients reported higher satisfaction compared to other patients, which may reflect broader disparities in healthcare experiences or expectations and warrants further qualitative investigation. Additionally, the strong association between temporary crowns and increased satisfaction across all parameters underscores their value not only in soft tissue shaping but also in enhancing patient-perceived esthetics and function.

The overall high levels of patient satisfaction observed in this study are consistent with prior literature indicating that implant therapy typically meets or exceeds patients’ expectations for esthetics and function. Korfage et al. (2018) [25] systematically reviewed 16 studies and reported that patients generally have high pre-treatment expectations, prioritizing functional improvement followed by esthetics. The review also noted that women tend to report higher expectations than men and that cost remains a major deterrent. Our findings, demonstrating over 80% of patients rating their outcomes between 8 and 10 for all satisfaction domains, suggest that the AEGD program successfully met these expectations through comprehensive care and interdisciplinary collaboration.

The findings of this study provide empirical evidence supporting the effectiveness of structured implant training in AEGD programs. They validate previous smaller-scale reports by Starr and Maksoud [10,11], and suggest that general dentistry residents, when properly supervised and calibrated, can deliver implant outcomes on par with those of specialists. Furthermore, the identification of modifiable risk factors such as plaque control and the use of provisional restorations can inform enhancements to the educational curriculum and clinical protocols.

Our dataset only included implants that had survived at least one year and were present at recall visits, which inherently excludes cases of early implant failure. Early failures are defined as implants that do not achieve or maintain osseointegration prior to functional loading, often due to poor healing, infection, or inadequate primary stability (Albrektsson et al., 1986 [9]; Esposito et al., 1998 [26]). As such, our findings cannot account for implants that were removed prior to recall, which may also explain the absence of severe peri-implantitis cases in our cohort.

The favorable outcomes observed in this study must be interpreted within the context of the structured educational environment in which implants were placed. Contemporary training in implant dentistry increasingly incorporates simulation-based learning, cadaveric dissection experiences, and blended educational approaches to improve surgical competence and reduce early complications. The recent literature [27,28] highlights that structured training environments and deliberate practice models may shorten learning curves and enhance procedural predictability. However, variability in case selection, supervision intensity, and cumulative surgical experience are likely to also contribute to clinical outcomes.

## 6. Limitations

This study is limited by its retrospective design, including the lack of randomization or blinding, as well as potential variability in residents’ experience, surgical technique, and follow-up duration. Self-reported satisfaction data may be subject to subjective bias. Implant failures and removals, which occurred in 2.28% of cases, were identified through billing records, but the specific clinical causes were not analyzed, limiting interpretation of underlying etiologic factors such as surgical, prosthetic, or patient-related contributors. In some outcome categories, regression models yielded large odds ratios with wide confidence intervals, likely reflecting sparse data bias or quasi-complete separation; these associations should therefore be interpreted with caution. Therefore, the outcome represents implant retention at recall rather than a true time-to-event survival rate, and comparisons with survival rates from other studies may not be methodologically equivalent. Future prospective studies with detailed failure analyses are warranted to validate these findings and inform strategies to improve long-term implant success.

## 7. Conclusions

Within the limitations of this retrospective study, dental implants that remained in function for at least one year after being placed and restored by AEGD residents under structured supervision demonstrated high satisfaction rates comparable to those reported in the general literature. The low incidence of biological and technical complications underscores the effectiveness of comprehensive clinical training and faculty-guided protocols in ensuring successful outcomes. These findings highlight the capacity of postdoctoral general dentistry programs to achieve predictable implant performance and contribute meaningfully to both patient care and resident competency development.

## Figures and Tables

**Table 1:** Distribution demographics and health condition (Charts n = 262).

Characteristic	Frequency (%)
**Gender**	
**Female**	138 (52.7%)
**Male**	124 (47.3%)
**Race**	
**White**	153 (58.4%)
**African American**	29 (11.1%)
**Unknown**	67 (25.6%)
**Asian**	7 (2.7%)
**Native American**	6 (2.3%)
**Ethnicity**	
**Not Hispanic**	201 (76.7%)
**Hispanic**	30 (11.5%)
**Unknown**	31 (11.8%)
**Common Health Concerns**	
**High blood pressure**	75 (28.6%)
**Alcoholism**	69 (26.3%)
**Diabetes**	60 (22.9%)
**No conditions**	48 (18.3%)
**Tobacco use (smoking/chewing)**	48 (18.3%)
**Arthritis**	43 (16.4%)
**Penicillin allergy**	38 (14.5%)
**No known allergy**	150 (57.3%)

**Table 2:** Overall follow-up duration.

Variable	Value
**Total implants**	262
**Mean follow-up (years)**	4.19 ± 1.95
**Median follow-up (years)**	3.85
**Range (years)**	1.15–9.78
**Follow-up Duration (Years)**	***n* (%)**
1–<2 years	42 (16.0%)
2–<3 years	73 (27.9%)
3–<4 years	47 (17.9%)
4–<5 years	30 (11.5%)
5–<6 years	21 (8.0%)
6–<7 years	25 (9.5%)
7–<8 years	14 (5.3%)
8–<9 years	8 (3.1%)
≥9 years	2 (0.8%)

**Table 3:** Implant-crown-related complications outcome (n = 262).

Outcome	Yes (%)	No (%)	Unsure (%)
**Implant-Related**			
Early peri-implantitis	30 (11.45%)	231 (88.17%)	N/A
Moderate peri-implantitis	1 (0.38%)	231 (88.17%)	N/A
Abutment fracture	1 (0.38%)	259 (98.85%)	2 (0.76%)
Screw fracture	1 (0.38%)	258 (98.47%)	3 (1.14%)
Screw loosening	22 (8.39%)	236 (90.07%)	4 (1.52%)
**Crown-Related**			
Loosening in cement seal	9 (3.43%)	248 (94.65%)	5 (1.90%)
Crown fracture	2 (0.76%)	257 (98.09%)	3 (1.14%)
Chipping of crown	9 (3.43%)	249 (95.03%)	4 (1.52%)
**Prosthesis**	**Alpha ^1^ (%)**	**Bravo ^2^ (%)**	**Charlie ^3^ (%)**
Marginal adaptation	246 (93.89%)	16 (6.11%)	0 (0.0%)
Anatomical form	239 (91.22%)	23 (8.78%)	0 (0.0%)
Color match	243 (92.75%)	17 (6.49%)	2 (0.76%)
Occlusal wear	260 (99.24%)	1 (0.38%)	1 (0.38%)

^1^ Alpha scores represented ideal clinical conditions. ^2^ Bravo indicated minor deviations such as slight contour or color mismatch. ^3^ Charlie reflected major defects including visible fractures or pronounced color discrepancies.

## Data Availability

The original contributions presented in this study are included in the article. Further inquiries can be directed to the corresponding author.
